# Supplementary material for: A data reduction and compression description for high throughput time-resolved electron microscopy
Source: Nat Commun. 2021 Jan 28;12:664. doi: 10.1038/s41467-020-20694-z (PMC7844242; doi:10.1038/s41467-020-20694-z)
Supplement: Supplementary file 1 — Supplementary Information [file 41467_2020_20694_MOESM1_ESM.pdf]

Supplementary Information for
"A Data Reduction and Compression Description for High Throughput Time-Resolved Electron Microscopy"

Abhik Datta[1,2], Kian Fong Ng[1,2], Deepan Balakrishnan[1,2], Melissa Ding[3], See Wee Chee[1,2,4], Yvonne Ban[1,2], Jian Shi[1,2], N. Duane Loh[1,2,4]

[1]Centre for BioImaging Sciences, National University of Singapore, Singapore 117557.
[2]Department of Biological Sciences, National University of Singapore, Singapore 117557.
[3]Department of Computer Science and Engineering, Ohio State University, Columbus, OH 43210, USA.
[4]Department of Physics, National University of Singapore, Singapore 117551.

Table of Contents

ReCoDe Data Format 1
Supplementary Method 1: The ReCoDe Data Format 1
Data Compression 6
Supplementary Figure 2: Decompression Throughput 6
Supplementary Discussion 3: Single-Threaded Write Performance 6
Supplementary Discussion 4: Effect of Data Reduction on Image Quality 7
Supplementary Discussion 5: Coincidence Loss Estimation 8
Supplementary Discussion 6: Storage Requirements of Cryo-EM Experiments with Movie-mode Detectors 10
Supplementary Figure 7: Comparison of ReCoDe and MRCZ 11
Supplementary Figure 8: Shape and Size Distribution of Secondary Electron Puddles 13
Supplementary Figure 9: Compressibility of Representations of Centroids 14
Signal-Noise Calibration 15
Supplementary Figure 10: DE-16 detector ADU distribution 15
Supplementary Note 11: On-the-fly Signal-Noise Calibration 16
Supplementary Note 12: Fine Signal-Noise Calibration for the DE-16 Detector 17
Supplementary Note 13: Estimating Backscattering 23
References 26

ReCoDe Data Format

Supplementary Method 1: The ReCoDe Data Format

Reduced Compressed Data Format

All intermediate and merged ReCoDe files begin with a ReCoDe header which has a fixed length (512 bytes) and a static structure (described in Supplementary Table 1). The headers are followed by an optional section (non-standard frame metadata descriptions) that lists names and sizes of additional fields, such as timepoint or scan position, in each frame's metadata. This is followed by the header of the original file (such as MRCS or Sequence headers). In merged files, these are followed by a frame metadata section that stores the compressed sizes of frame data as well as the values of additional non-standard frame metadata. This section is followed by the actual reduced compressed frame data. The merged ReCoDe file structure is described in Supplementary Fig. 1a. Optionally, the per-pixel calibration data, used for signal-noise separation, can be appended at the end of the file as a single frame. The availability of this frame is indicated in the ReCoDe header field "Is Calibration Data Appended" (see Supplementary Table 1).

The intermediate files generated during multithreaded reduction compression are optimized for sequential access and have a slightly different structure than merged ReCoDe files. In intermediate files, the ReCoDe header, the non-standard frame metadata descriptions, and the original file's header are followed by frame-blocks, with each frame-block containing a single frame's metadata followed by the actual reduced compressed data for the frame. The intermediate ReCoDe file structure is described in Supplementary Fig. 1b.

The exact frame metadata information and the reduced compressed information retained for each frame depends on the reduction level. For example, in L1 the metadata for each frame contains three 4-byte-long numbers: the size of the compressed binary image, the size of the compressed pixel intensities, and the number of foreground pixels. Retaining the size of compressed data in each frame is necessary as the compressed sizes vary. Storing these sizes in the metadata block, which appears before the actual compressed frame data in the merged file, allows efficient random access of frames in merged files by only parsing the metadata block. In contrast, the intermediate files are designed for sequential data access. Decompressing the pixel intensity values requires knowing the size of the decompressed data, therefore storing the number of foreground pixels explicitly rather than inferring it from the decompressed binary map, allows the binary map and the pixel intensity values to be decompressed in parallel. The frame metadata section in intermediate files additionally stores frame ids. Since frames may not be distributed to intermediate files in any specific order, frame ids are used to ensure that frames are placed sequentially when creating the merged file in the absence of other optional frame ordering information such as timepoints.

a

**Non-standard Frame Metadata Descriptions (Optional)**	**Size (Bytes)**
Field 1 Name	99
Size of Field 1 (bytes)	1
⋮	
Field ***N****** Name	99
Size of Field ***N*** (bytes)	1

****N***: The number of non-standard metadata fields is specified in the header

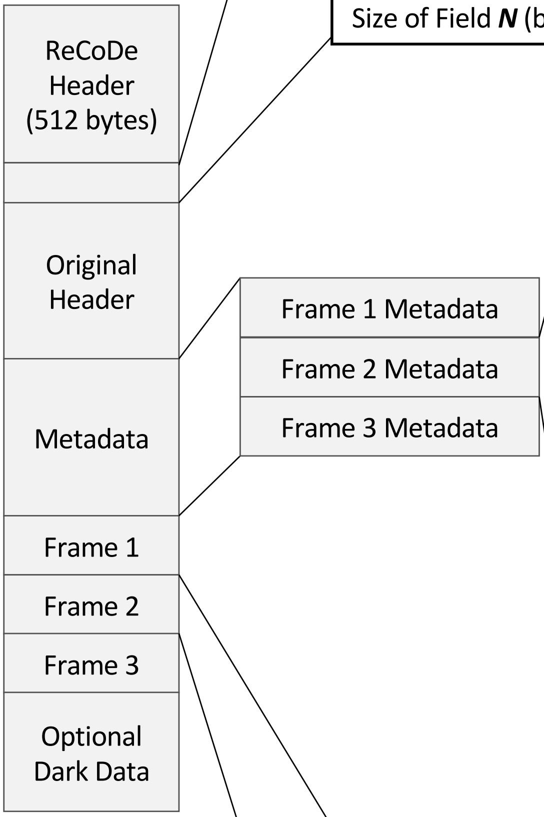

**Frame Metadata Information (per Frame)**	**Size (Bytes)**			
	**L1**	**L2**	**L3**	**L4**
Size (Bytes) of Compressed Binary Map	4	4	4	4
Size (Bytes) of Compressed Pixel Intensities	4			
Number of Pixel Intensities Stored	4			
Size (Bytes) of Compressed Puddle Summary Statistics		4		
Number of Secondary Electron Puddles		4		
Non-standard Frame Metadata (Optional)	Depends on the number (***N***) and sizes of fields described in table above			

**Frame Data (per Frame)**	**L1**	**L2**	**L3**	**L4**
Compressed Binary Map	●	●	●	●
Compressed Pixel Intensities	●			
Compressed Puddle Summary Statistics		●		

b

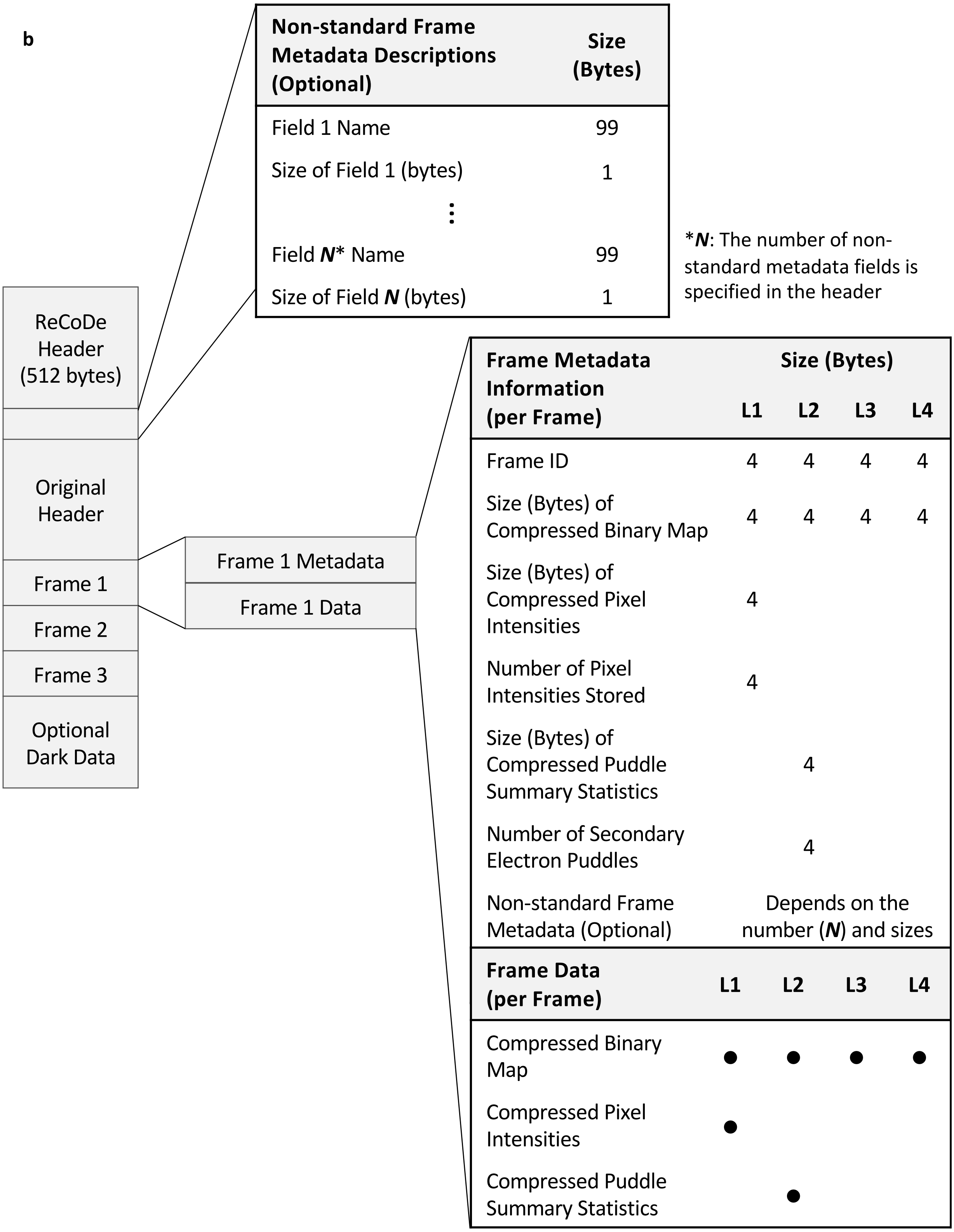

Supplementary Figure 1: ReCoDe file structure for a time series with only 3 frames. **(a)** Structure of merged ReCoDe file and **(b)** structure of intermediate ReCoDe files.

Supplementary Table 1: Structure of ReCoDe header.

Field	Size (bytes)	Possible Values (Code = Value) / Description
Unique Identifier	8	Always 158966344846346
Major Version	1	
Minor Version	1	
Is Intermediate File	1	1 = Intermediate File, 0 = Merged File
Reduction Level	1	1 - 4
Is Compressed	1	0 = Reduction Only, 1 = Reduction + Compression
Is Bit-packed	1	0 = False., 1 = True
Target Intensity Bit-depth	1	Bit-depth of output (ReCoDe) data
Number of Rows In a Frame	4	
Number of Columns in a Frame	4	
Number of Frames	4	
Frame Metadata Size	1	Size of each frame's metadata in bytes
Number of Non-standard Frame Metadata Fields	1	No. of additional frame specific information (time point, scan position, etc.)
Secondary Electron Puddle Statistics	1	0 = Default (Max), 1 = Max, 2 = Sum. Used only in L2.
Secondary Electron Puddle Centroiding Scheme	1	0 = Default (Centre of Mass), 1 = Centre of Mass, 2 = Max. Pixel, 3 = Central Pixel. Used only in L2 and L4.
Compression Method	1	0 = Deflate, 1 = bzip, 2 = LZMA, 3 = Snappy, 4 = Lz4
Compression Level	1	1 – 9. Internal optimization level of compression algorithms
Original File Type	1	0 = Binary, 1 = MRC, 2 = Sequence, 255 = Others
Original File Header Size	2	1024 for MRCS and Sequence, 0 otherwise
Original File Header Position	1	Always 1 (indicating after ReCoDe header). The option "before ReCoDe header" is deprecated in version 0.2.
Original Source File Name	100	
Dark Noise File Name	100	
Signal-Noise Calibration Parameter (S)	8	See signal-noise calibration algorithm for details. Assumed to have the same data type as source data.
Is Calibration Data Appended	1	0 = False, 1 = True
Frame Offset	4	Index of First Used Frame in Original Data
Dark Frame Offset	4	Index of First Used Frame in Calibration Data
No. of Dark Frames Used for Calibration	4	
Source Bit-depth	1	Bit-depth of original data. Can be different from output bit-depth, if scaling is used.
Source Data Type	1	0 = Unsigned Integer, 1 = Signed Integer, 2 = Float
Target Data Type	1	Data type of output (ReCoDe) data. 0 = Unsigned Integer, 1 = Signed Integer, 2 = Float
Checksum	32	
Reserved For Future Use	219	

Reduced Data Format

ReCoDe also has the provision of only reducing the data without compressing it. The reduced data files (merged and intermediate) follow the same structure as the corresponding reduced and compressed ReCoDe files. However, the frame metadata sections for L1 and L2 reduction levels, stores the number of pixel intensities and the number of secondary electron puddles, respectively, in addition to any non-standard frame metadata. For L3 and L4 reduction levels, there is no standard metadata information, only non-standard frame metadata are stored if present. The frame data section stores the same information as in the reduction compression case but in uncompressed form.

Validation frames

ReCoDe also has the option of saving every n^{th} frame (where n is user-specified) simultaneously in an uncompressed unreduced raw format. These frames are saved as a separate MRC stack and a reduced-compressed version of this frame is still saved in the ReCoDe format. Comparing these two versions of the same frame can serve as a useful validation/diagnostic.

Merging Part Files

Frames processed by a reduction compression thread are sequentially appended to its intermediate file. If the frames processed by a reduction compression thread are time-ordered, i.e. the $(i-1)^{th}$ frame precedes the ith frame in time, the merging step becomes trivial. Merging k intermediate files require maintaining a pointer to the earliest unmerged frame in each intermediate file. The merging involves finding the earliest frame among the frames pointed to by the k pointers (say the ith frame of the nth intermediate file), adding that frame to the merged file, and moving the nth pointer to the next frame in the intermediate file. This merging accesses the intermediate files and the merged output file sequentially and is therefore very fast.

Data Compression

Supplementary Figure 2: Decompression Throughput

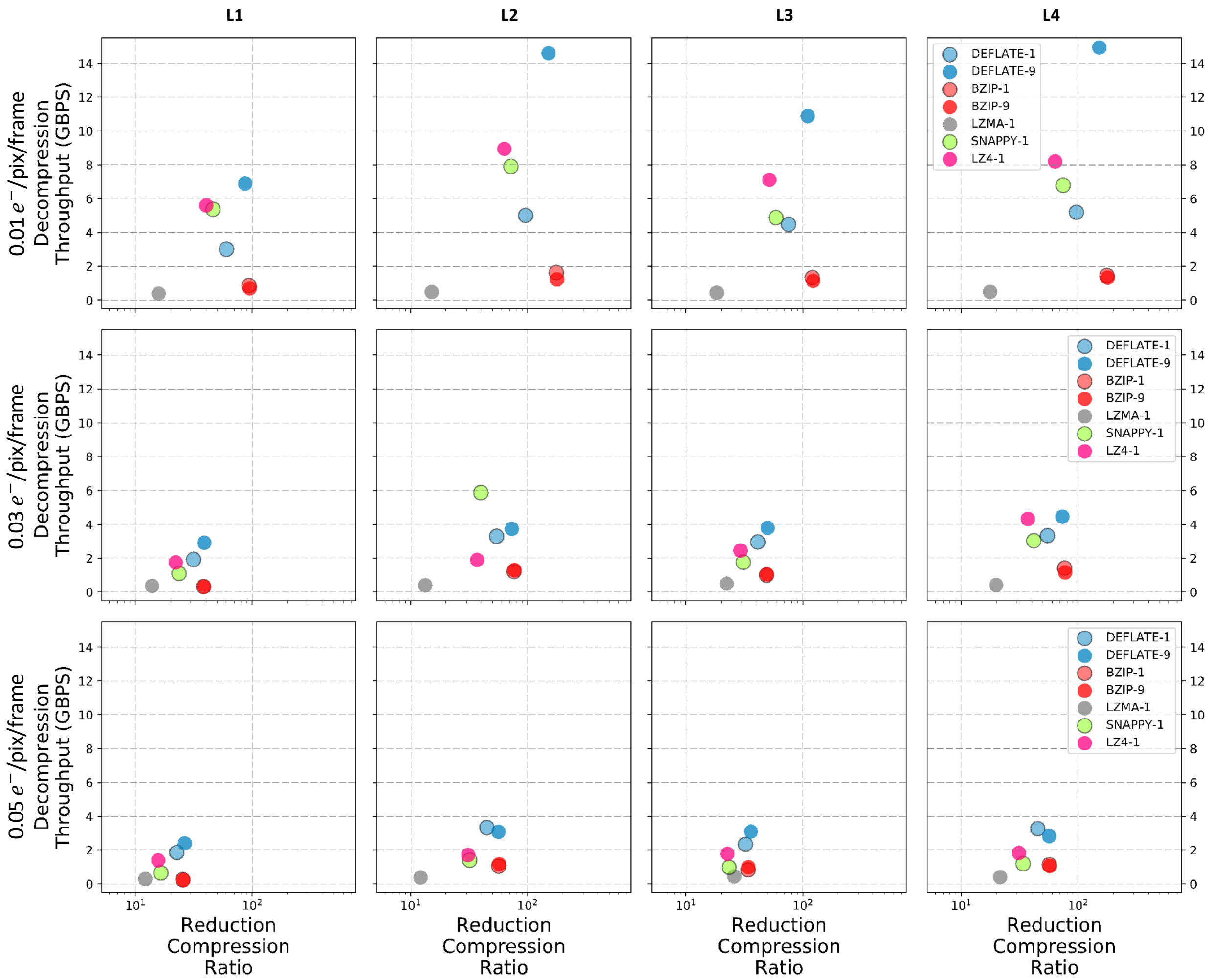

Supplementary Figure 2: Decompression throughputs. The reduction compression ratios and decompression throughputs of five algorithms: Deflate, bzip2, LZ4, LZMA and SNAPPY. Here, the reduction compression ratio is the ratio between the sizes of the original (uncompressed) data and the reduced compressed data. Suffix -1 and -9 refer to internal optimization levels of the algorithms corresponding to the fastest compression and the optimal compression, respectively. For LZ4, LZMA and SNAPPY the internal optimizations produced similar results, hence only the default level is shown. The three rows of scatter plots correspond to three different electron fluxes: 0.01, 0.03 and 0.05 e/pixel/frame from top to bottom, and the four columns of scatter plots correspond to the four reduction levels: L1 to L4 from left to right. The throughputs are based on single threaded operation of ReCoDe and include the time taken for both reduction and decompression.

Supplementary Discussion 3: Single-Threaded Write Performance

In the absence of distributed storage servers, on-the-fly reduction compression will have to write to standard hard drives or SSDs, which support a limited number of simultaneous writes. To evaluate ReCoDe's on-the-fly reduction compression performance in such cases, we evaluated an alternative implementation, where the reduction compression threads do not independently write to their respective intermediate files. Instead, a single offloader thread performs all writes to disk. This offloader thread is invoked by the reduction-compression threads when their local buffers are full. The reduction compression threads then have to wait for the offloader thread to attend to their requests and clear the buffer. This implementation emulates the worst-case write performance,

where a single thread sequentially accesses the disk. The experiment was repeated for four flux levels (Supplementary Fig. 3). From the observed speed-ups, we estimate, based on Amdahl's law[1], that even with one offloader thread ~89% of the code runs in parallel.

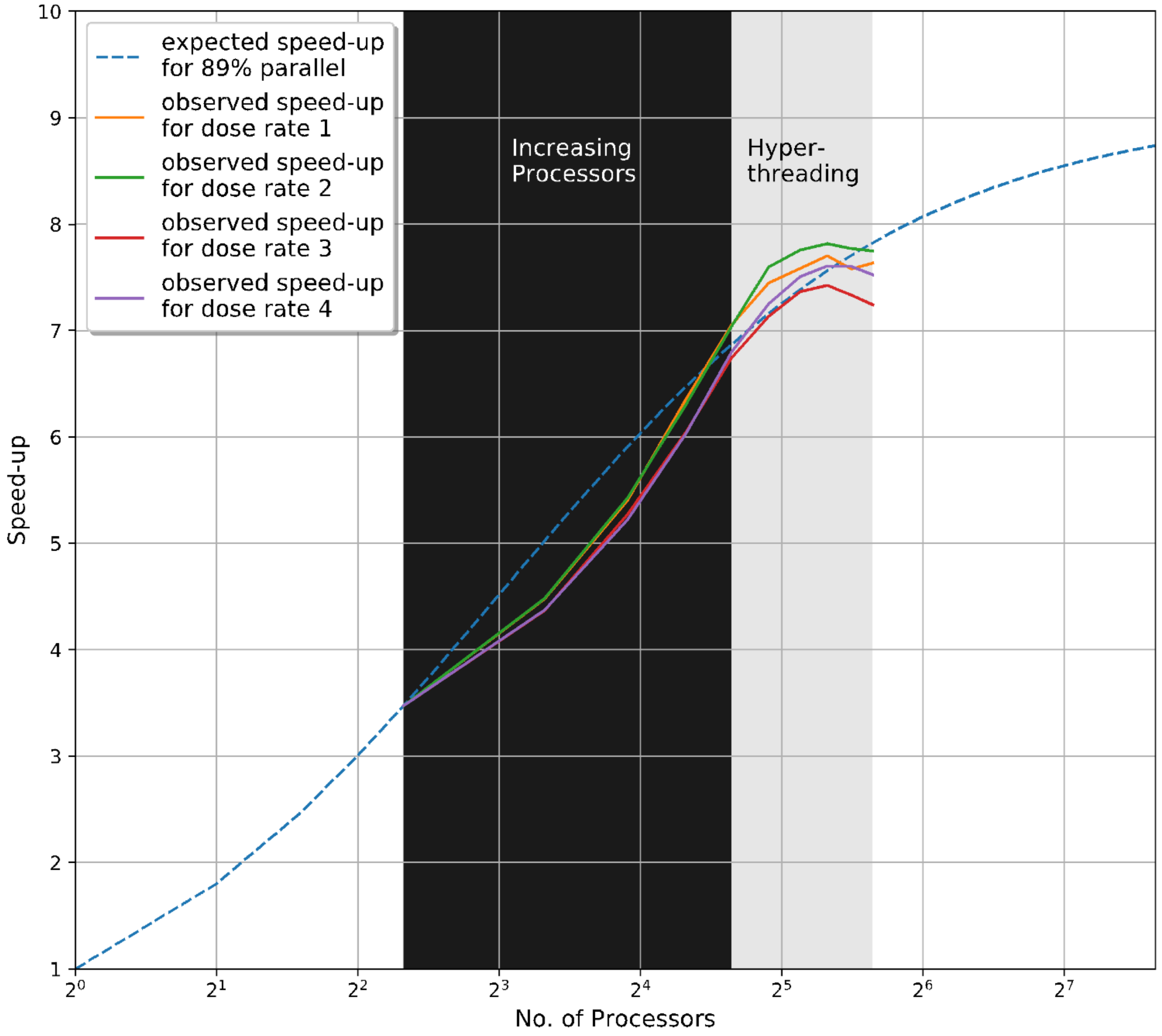

Supplementary Figure 3: Single threaded write performance of ReCoDe. The solid lines show the observed speed-ups with increasing processors for four flux levels: 0.005, 0.014, 0.027 and 0.034 e/pixel/frame. The blue dashed line shows the predicted speed-up for an 89% parallel code, as per Amdahl's law[1]. The simulations were performed on a single compute node with 28-cores (14 core x 2 chips), 2.6 GHz Intel Xeon processors and 512 GB RAM. Simulations using greater than 28 threads used hyperthreading.

Supplementary Discussion 4: Effect of Data Reduction on Image Quality

A knife-edge test was performed using a beam blanker, with an electron flux of ~0.8 e/pixel/s using the DE-16 detector operating at 400 fps exposed for 50 seconds, resulting in a total dose of ~40 e/pixel and an effective electron flux of *~0.002*e/pixel/frame across 20,000 frames. Each frame was resampled to twice the resolution using bicubic interpolation and counting was performed on the resampled frames following one of the three localization strategies: for each electron puddle, we either localized to the weighted centroid, non-weighted centroid, or the pixel with the maximum value. The straight edge of the beam blanker was visually determined in the counted image obtained by summing the counted frames. The edge spread function (ESF) was estimated from the average of multiple one-dimensional profiles measured along the normal to the straight edge. The edge spread function was differentiated to give the line spread function (LSF) which was Fourier transformed to get the MTF.

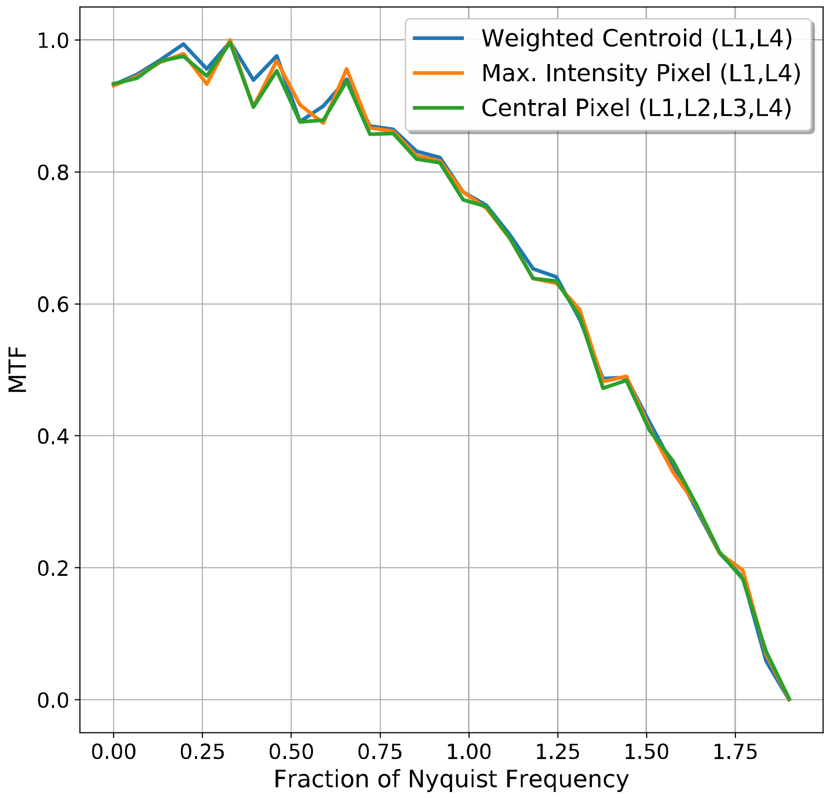

Supplementary Figure 4: Data reduction and image quality. MTFs estimated using the knife-edge method from three counted images of the beam blanker are shown. The three counted images are obtained using three different approaches to estimating the entry point of electrons from their secondary electron puddles (see Methods section for implementation details). For this MTF calculation a Dirac delta function is used as the PSF (instead of the actual detector PSF) to highlight the differences due to localization errors alone.

Supplementary Discussion 5: Coincidence Loss Estimation

To understand the role of puddle shape and size in coincidence loss estimation, five coincidence loss estimation strategies were evaluated (Table 1 of Main text). The first strategy assumes all secondary electron puddles to be of the same shape and size: 3×3pixels. With these assumptions, simulated images were used to calculate the actual frequency of puddle overlaps at twelve different dose rates. Similarly, in the second and third strategies puddle of sizes 2×2 pixels and 1 pixel were used to simulate images, respectively. In the fourth strategy, the size distribution of puddles was learned from actual DE-16 detector data, collected at very low dose rates (0.001 e/pixel/frame). The puddles were, however, assumed to be circular and the expected coincidence loss was analytically computed. The final strategy was to simulate images following the learned shape as well as size distributions of the puddle and compute the actual frequency of puddle overlaps. The results indicate that shape and size information is critical in estimating coincidence loss, without which, coincidence loss values are underestimated (third and fourth columns compared to the fifth column in Table 1 of Main text).

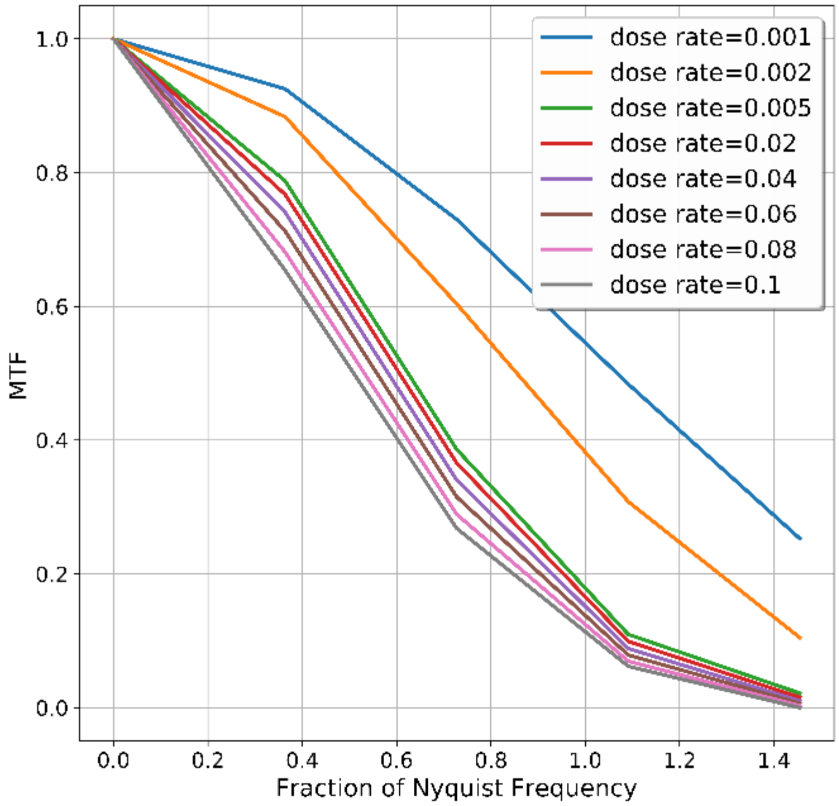

Supplementary Figure 5: Effects of coincidence loss on counting accuracy. MTFs corresponding to counted images, simulate at electron fluxes ranging from 0.005 to 0.1 $e/Å^2/s$ are shown. As electron flux increases, the MTF of counted images decrease due to higher coincidence loss, particularly at higher frequencies. The counting used here reduces each puddle to its weighted centroid pixel.

Supplementary Discussion 6: Storage Requirements of Cryo-EM Experiments with Movie-mode Detectors

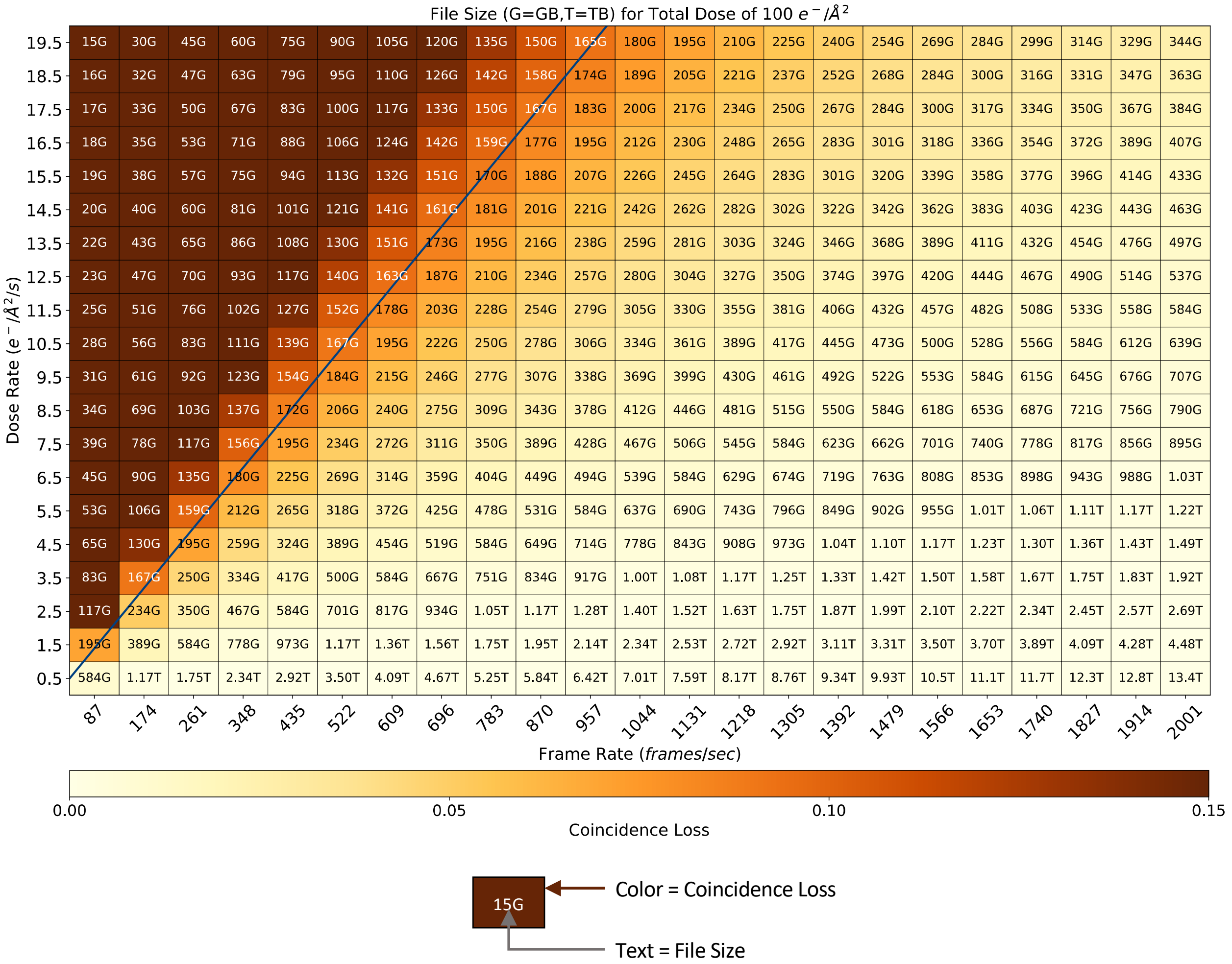

Supplementary Figure 6: Storage requirements of cryo-EM experiments with movie-mode detectors. Shows how lower dose rates and higher temporal resolutions require collecting prohibitively large amounts of data in the absence of reduction compression. The text in each cell indicates the amount of data needed to achieve a total dose of 100 $e/Å^2/s$, across different electron fluxes and temporal resolutions. This calculation assumes a 4096×4096 pixel detector producing 16-bit images at a magnification with pixel size equal to *1Å*. The cell colors represent the coincidence loss suffered at that electron flux and temporal resolution.

Supplementary Figure 7: Comparison of ReCoDe and MRCZ

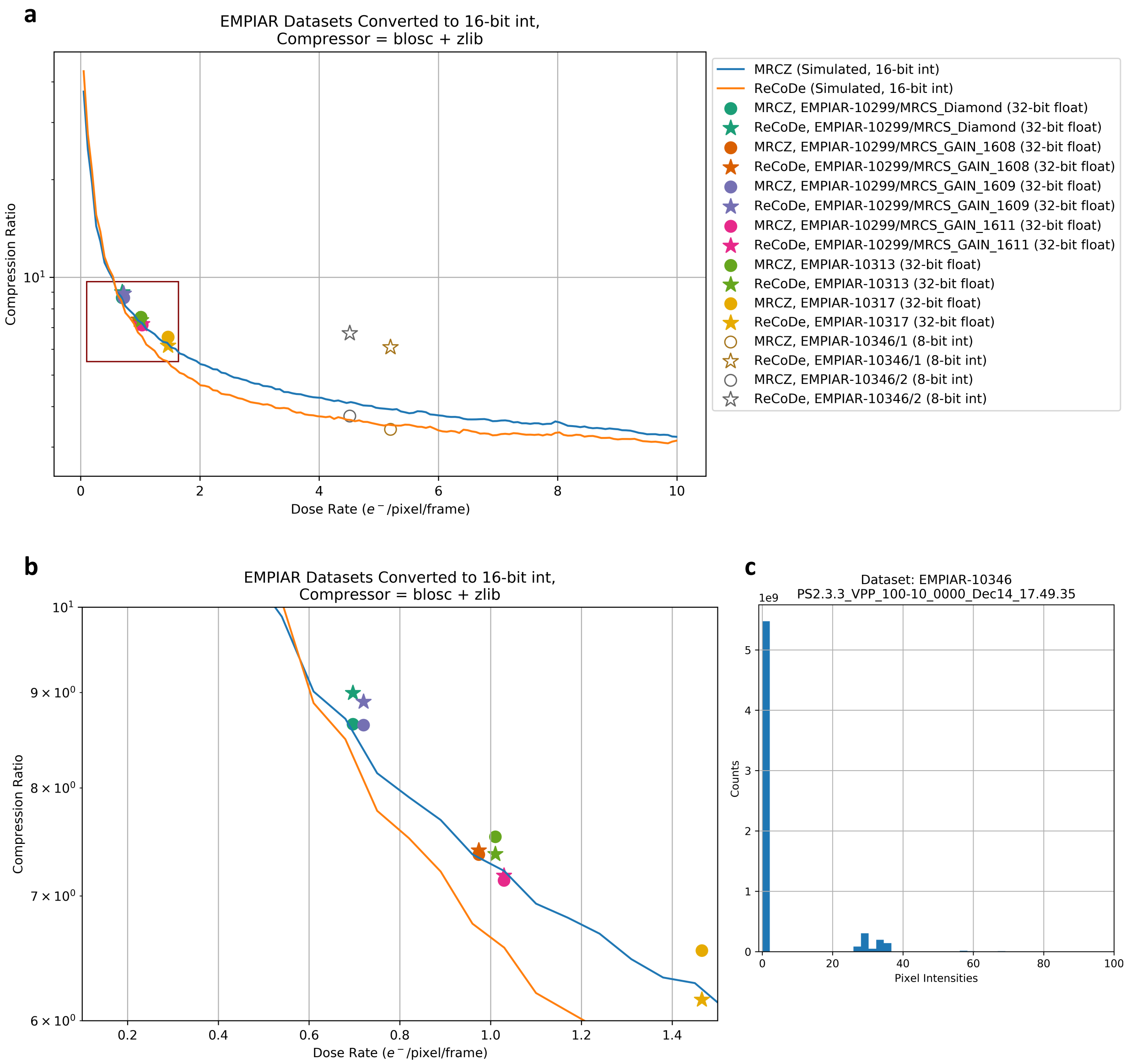

Supplementary Figure 7: Joint comparison of ReCoDe and MRCZ using simulated and EMPIAR datasets. (a) shows compression ratios achieved using ReCoDe and MRCZ on simulated and EMPIAR datasets. The simulated datasets had 16-bit unsigned integer data and for the purpose of standardisation, the EMPIAR datasets were also converted to 16-bit unsigned integer. The electron events per pixel follows a Poisson distribution in the simulated datasets. The underlying compression algorithms used for this comparison is Blosc + zlib (Deflate) for both MRCZ and ReCoDe. For the simulated data ReCoDe achieves higher compression ratios than MRCZ at lower dose rates (< 0.58 e/pix/frame). In case of EMPIAR datasets with 32-bit floating point data, ReCoDe gives slightly better compression than MRCZ up to dose rates <1 e/pix/frame. (b) shows the highlighted region of (a), containing the data point for the 32-bit floating point datasets, in greater detail. The crossover point for compression ratios achieved by ReCoDe and MRCZ can be seen to be approximately at a dose rate of 0.58 e/pix/frame for the simulated data. Whereas the crossover point for the EMPIAR datasets is closer to the dose rate of ~1.0 e/pix/frame, suggesting that electron events per pixel in the EMPIAR datasets deviate from the Poisson distribution and that these datasets are sparser than the simulated data. Note that the floating point data was converted to unsigned integer by normalising the pixel values to the 0-4096 range and rounding to the nearest integer. For the two datasets from EMPIAR-10346, that had 8-bit unsigned integer data, ReCoDe achieves much higher levels of compression than MRCZ, even though these datasets have a higher average dose rate. This is because these datasets contain sparse, high contrast frames. (c) shows the distribution of pixel intensities for one of the EMPIAR-10346 dataset, acquired using a Volta phase

plate. The histogram shows that the data is highly sparse with very high frequency of zeros and a small cluster of high count pixels, leading to an overall high average dose rate. Such sparse data enables ReCoDe to achieve higher compression ratios. Overall, these comparisons suggest that for various types of real datasets, ReCoDe can be an effective choice for data archival.

Supplementary Figure 8: Shape and Size Distribution of Secondary Electron Puddles

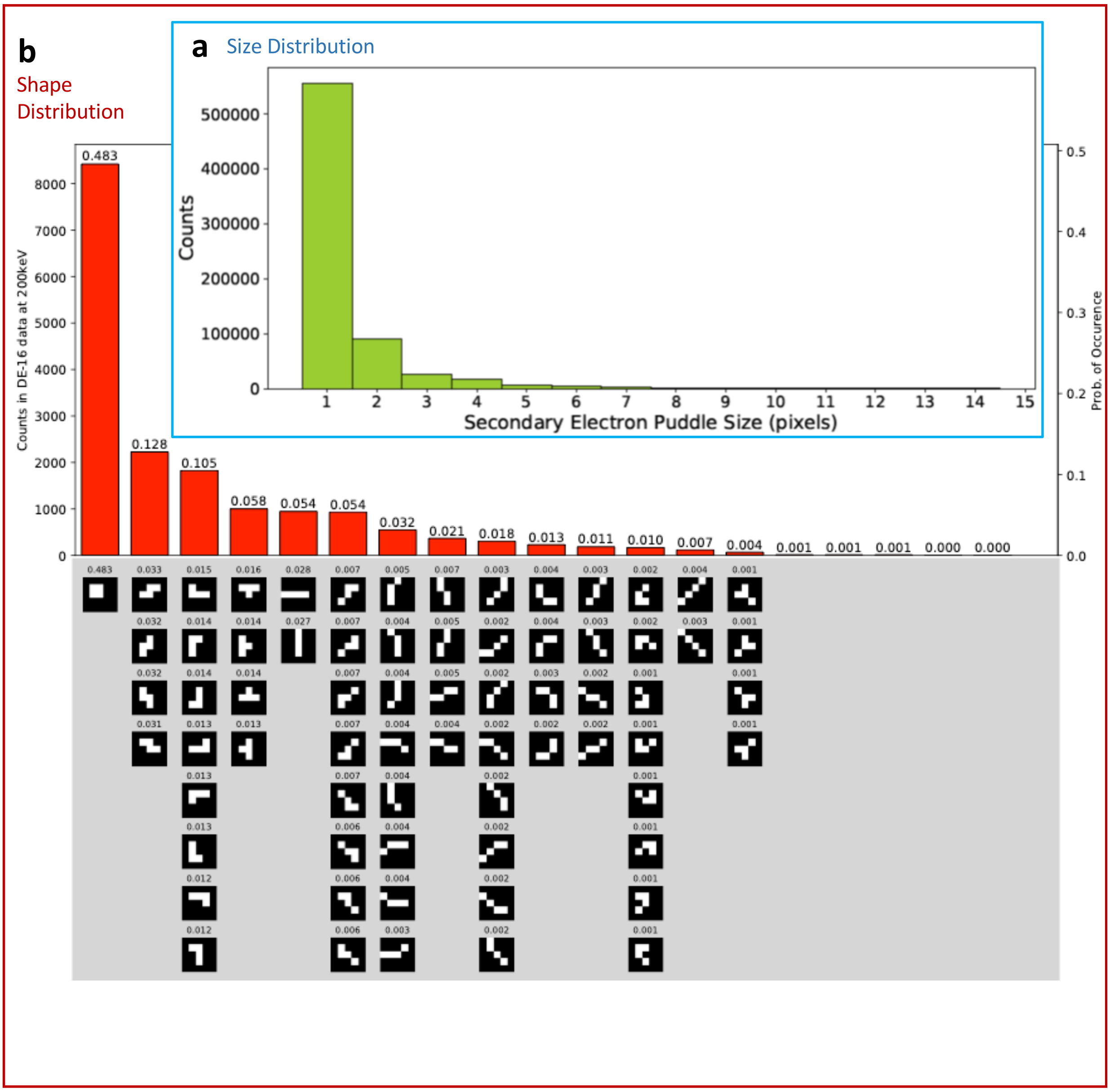

Supplementary Figure 8: Shape and size distribution of DE-16 secondary electron puddles. **(a)** The size distribution of 706,217 secondary electron puddles extracted from a DE-16 dataset acquired at a dose rate of 0.001 e^{-}/pixel/sec. **(b)** Frequency of all possible rotational variants of electron puddles with an area of four pixels. The histogram tabulates the total number of counts for each unique four pixel puddle shape and its rotational variants and the corresponding probability of occurrence among the 17,419 four pixel sized electron puddles. Each bar in the plot represents one of the 16 unique shapes of four pixel sized puddles. Binarized representations of the puddle shapes, for orientations having occurrence probabilities higher than 0.001, are shown beneath. The shapes are sorted from left to right and then from top to bottom by their occurrence probabilities.

Supplementary Figure 9: Compressibility of Representations of Centroids

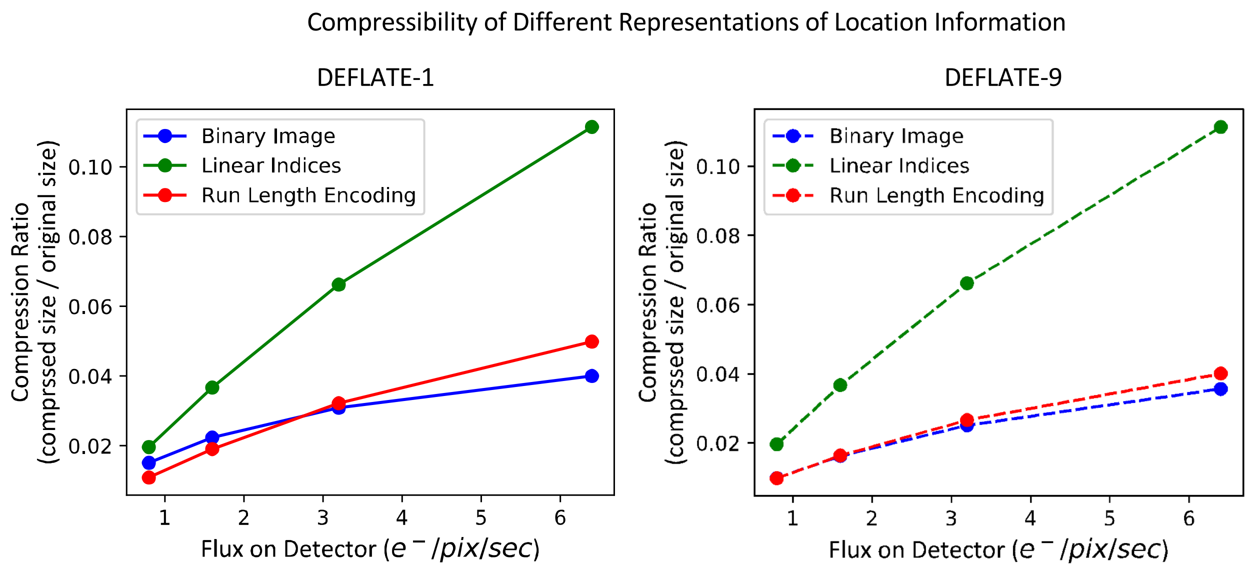

Supplementary Figure 9: Compressibility of three representations of centroids. Compression ratios due to three encodings of secondary electron puddle's locations when using **(a)** Deflate optimized for speed and **(b)** Deflate optimized for compression. In the Linear Indices encoding, a centroid's location is represented as a single 2n-bit linear index. In Run Length Encoding the linear indices are sorted and run-length encoded (RLE), since the ordering of centroids is inconsequential. In the Binary Image encoding, the centroids are represented as a binary image (similar to L4). RLE and binary image representations achieve comparable levels of compression and are much more compressible than linear indices. However, the binary image representation is more efficient than RLE, which requires a sorting. We therefore chose the binary image representation to encode the spatial information across all reduction levels of ReCoDe.

Signal-Noise Calibration

Supplementary Figure 10: DE-16 detector ADU distribution

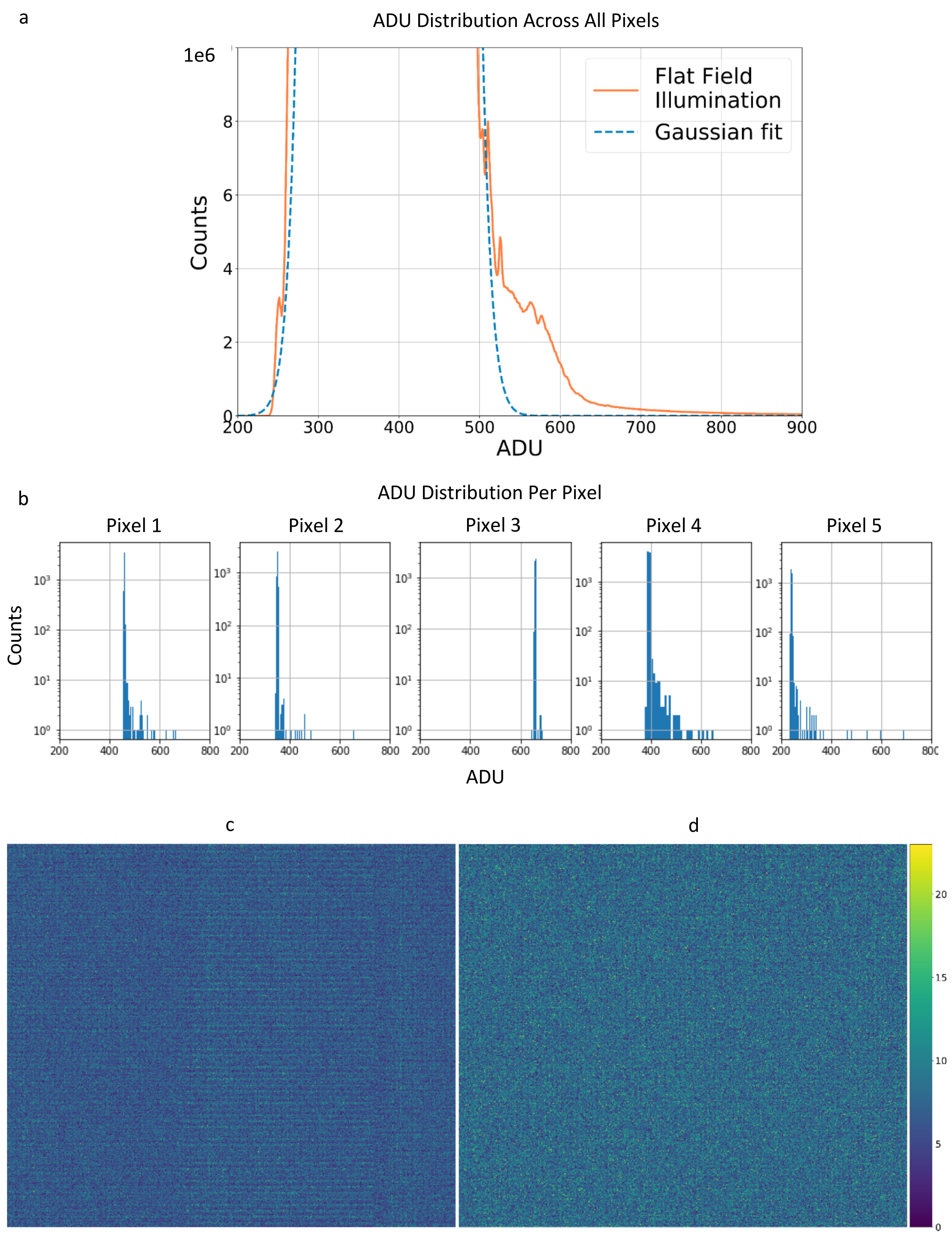

Supplementary Figure 10: DE-16 ADU distributions and adaptive calibration. **(a)** ADU distribution across all pixels in 10,000 frames of a low dose rate flat-field illumination frame-stack (0.001 e/pixel/frame), shows a smaller second distribution on the right due to actual electron events. **(b)** Visualizing the ADU distributions of individual pixels separately shows that pixels behave significantly differently from each other. **(c)** Using the same threshold for all pixels can severely bias counting. **(d)** The adaptive calibration used for DE-16 removes this bias.

Supplementary Note 11: On-the-fly Signal-Noise Calibration

Definitions:

r = the tolerable false positive rate in detecting electron events

R = stack of n flat field illuminated frames

$X_{i,R} = (x_i^0, \dots, x_i^n)$ be the values of the ith pixel across n frames of R

Signal-Noise Calibration Algorithm:

1. Do dark subtraction: $F = R - \widetilde{D}$, where $\widetilde{D} = \left[d_i = median(X_{i,R})\right]$
2. Fit a normal distribution $\mathcal{N}(x_i \mid \mu_F, \sigma_F)$ to F
3. Compute the global threshold τ, separating signal from noise, as $\tau = \mu_F + z * \sigma_F$, where z is given by:
$$r = P(Z \geq z) = \int_z^\infty \frac{1}{\sqrt{2\pi}} e^{\frac{-u^2}{2}} du$$
4. Select m random patches of size $k \times k$, such that k is odd and larger than the PSF's radius
5. Using F, identify outliers (signal), independently for each of the selected mk^2 pixels. Outliers in $X_{i,F}$: $\left\{x_i \mid x_i > \mu_{i,F} + z * \sigma_{i,F}\right\}$; assuming for the ith pixel $P(x_i) = \mathcal{N}\left(x_i \mid \mu_{i,F}, \sigma_{i,F}\right)$
6. For each patch, compute n_c: the number of connected components formed by the outlier pixels originating at the central pixel of the patch
7. Compute the expected dose per pixel $\bar{n}_c$ as the mean n_c across m patches
8. For the ith pixel, compute gain (g_i) as the median of the largest $\bar{n}_c$ values in $X_{i,F}$
9. The dark and gain corrected threshold for the ith pixel is:
$$\tau_i = \frac{\tau * \bar{g}}{g_i}$$

Supplementary Note 11: DE-16 On-the-fly signal-noise calibration algorithm for the DE-16 detector.

Supplementary Note 12: Fine Signal-Noise Calibration for the DE-16 Detector

We performed a detailed calibration process to compare the average dose obtained against that from ReCoDe's fast calibration. The fine calibration procedure implements a common mode correction and an area thresholding step, in addition to the previous calibration steps. We implemented both calibration processes on flat-field illuminated datasets with different incident electron dosage. Comparing the counts obtained, we aim to provide an error estimate on the false positives and quantify the speed-accuracy trade-off.

We caution that that the fine procedure here will vary substantially from device to device due to differences in the physical and electronics configuration of the detector.

Flat-field illuminated data frames were first collected at a sufficiently low dose rate that is comparable to the actual dose used for subsequent imaging. Next, we performed background noise subtraction by subtracting the median ADU value for each pixel. The median value is used since it provides a robust estimate of noise. With the dark subtracted dataset, we can proceed with a common-mode correction which reduces readout noise. To identify the common modes, the correlation between detector pixels must first be determined.

From a series of dark frames, collected in the absence of external illumination, we calculated the temporal correlations between pairs of pixels in a representative region of the detector. Next, we performed principal component analysis and transformed the pairwise correlation values, followed by K-means clustering. For the DE-16 detector, we identified correlations between pixels in every alternate column within each 1 by 256 block. The common-mode correction was therefore implemented block-wise per frame, with shared median values among the correlated pixels subtracted.

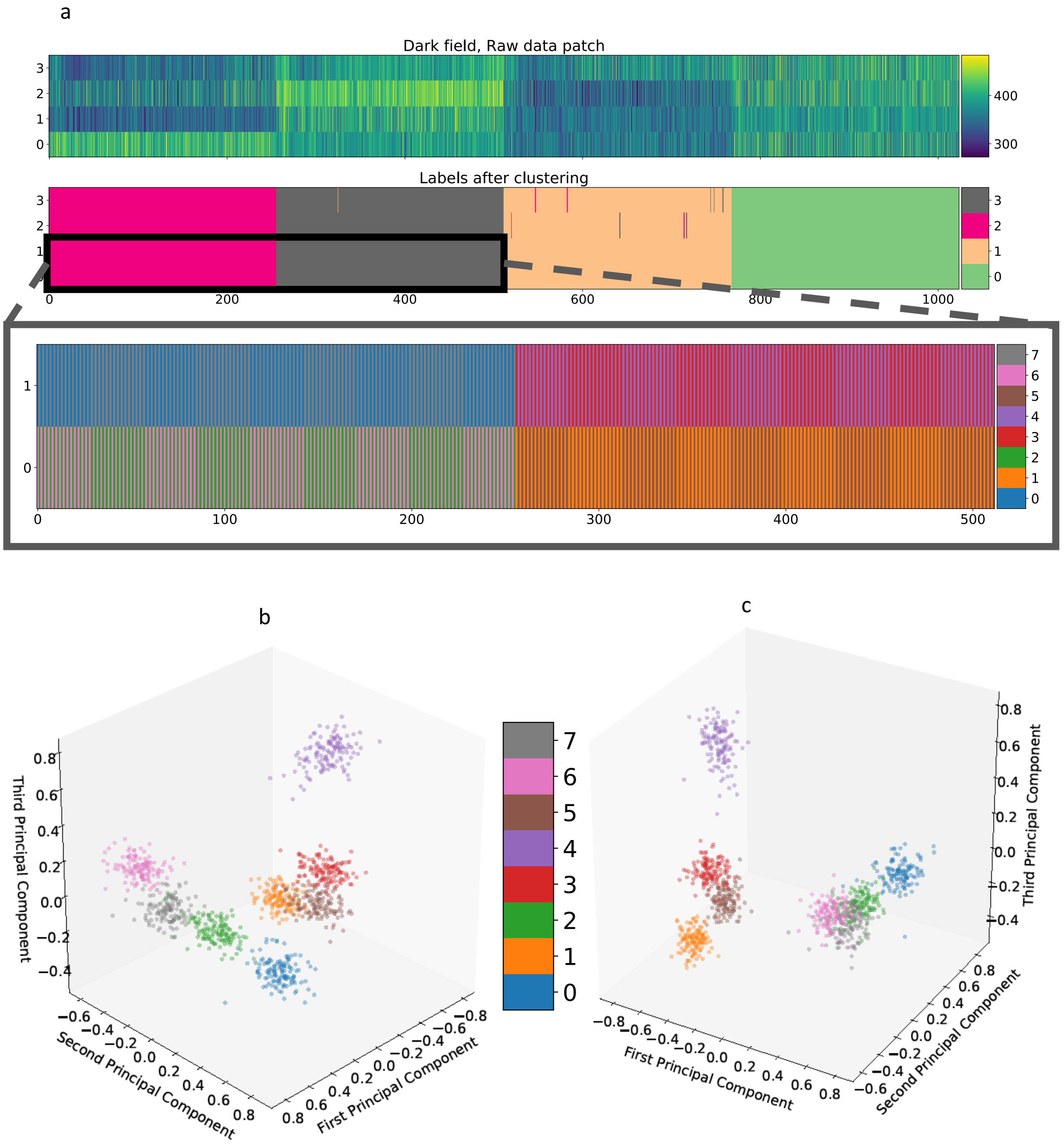

Supplementary Figure 12: Common mode correction for the DE-16 detector. (a) The topmost plot shows the response of 4 x 1024 pixel regions on the DE-16 detector in the absence of external illumination. From a series of dark frames, the pairwise temporal correlation between individual pixels, within the region was determined. The plot below shows the labels for the pixels after implementing principal component analysis (PCA), followed by k-means clustering on the pairwise correlation values. The pixels are grouped into four clusters, labelled with different colours. Pixels within each 4 x 256 block were observed to be grouped into the same cluster. The bottom plot shows the labels obtained for a 2 x 512 region spanning across two different blocks, after repeating the same process of PCA and k-means clustering with eight clusters. For each row of pixels, every alternate pixel across 256 columns was grouped into a single cluster. (b) k-means clustering of correlated pixels. The two 3D scatter plots show the transformed pairwise correlation values, for pixels in the 2 x 512 region, projected along the first, second and third principal components. Each data point represents a pixel, labelled according to its assigned cluster from k-means clustering. The same colour scheme is used to represent clusters as labels shown in the previous bottom plot. The plot on the left is a rotated view of the plot on the right, and together they show that all eight clusters are well separated from one another, with clear boundaries.

After the dark subtraction and common mode correction, we binned the ADU values of every pixel, across all frames, to construct a combined histogram. Assuming the dark non-signal values are distributed

normally, we can determine an appropriate threshold to separate signal from noise. The threshold is chosen based on the maximum acceptable false positive rate, at the expense of losing true electron counts.

For a selected subset of data frames, binary maps were created where only pixels with ADU values higher than the noise threshold are marked as signal pixels. Connected signal pixels with neighbors of 2-connectivity were identified. Here, a minimal area constraint is implemented to reduce the false positive rate. Connected signal pixels must have an area larger than the predetermined threshold to be considered as an electron puddle. We finally count the total number of such puddles per frame and calculate the average electron dose per pixel.

With the estimated average electron dose per pixel per frame, we can calculate the expected total dose for each pixel in the selected subset of data frames. The estimated number of electron events each pixel registers across the subset is used to estimate the detector's gain response per pixel. With the assumption that events with larger ADU values are more likely to be signal events than noise, we can determine an ADU threshold to separate them. For each pixel, we first ranked all detected events by their ADU values from the largest to smallest. Since we expect the number of signal events to be at least as many as the estimated number of electron events determined from the average dose, we identify the event with its rank matching the expected number of signal events and the next event with a lower rank and ADU value. The ADU threshold is then determined from the mean ADU value of both events. All other events with ADU lower than the threshold will be labeled as noise.

We can now perform the final step in the fine calibration process. With the gain threshold determined for every pixel, the counting process is repeated to calculate the actual number of electron events after gain correction. Similar to before, binary maps were created from pixels with ADU values higher than their respective gain thresholds. These signal pixels are marked and connected signal pixels with neighbors of 2-connectivity are identified as an electron puddle. The total number of electron puddles counted for each frame is the number of electron events for that frame. For the same selected subset of data frames, we now have a dark subtracted, common-mode and gain corrected average electron dose.

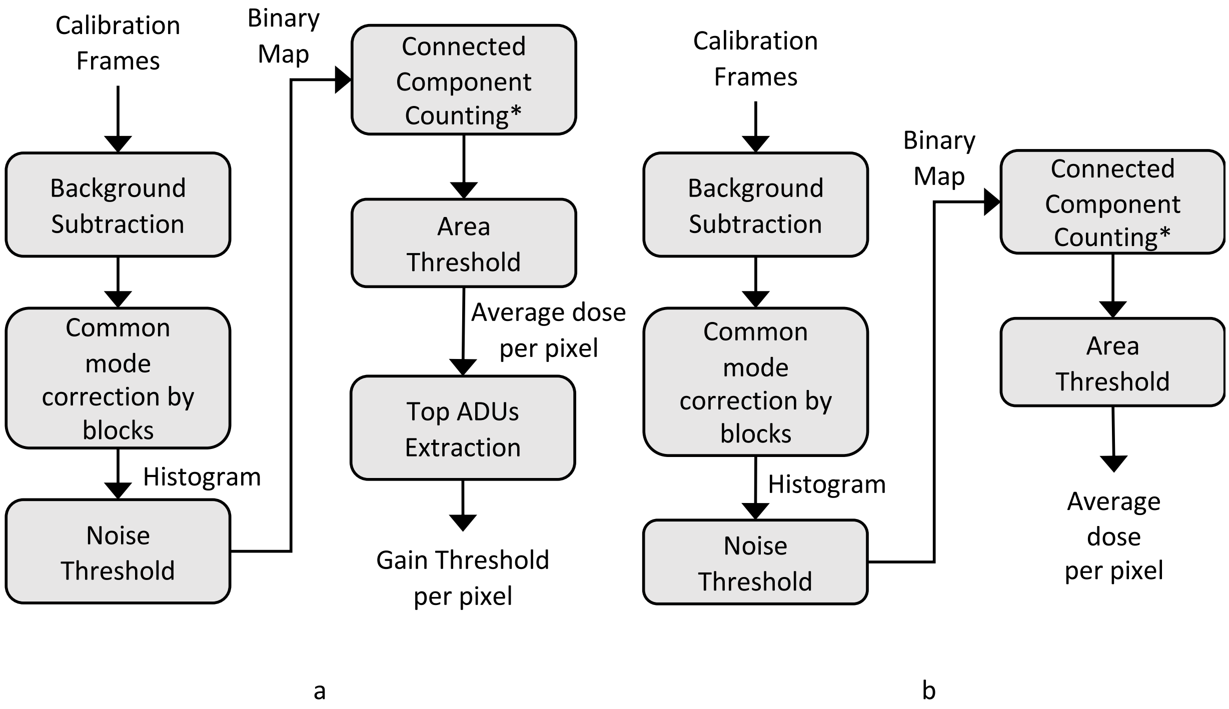

Supplementary Figure 13: Fine signal-noise calibration for DE-16 data. A variant of the on-the-fly calibration procedure described in Supplementary Figure 9 is presented here. We refer to this as a "fine calibration", as it incorporates common mode correction as well as an additional area threshold. After background noise subtraction across the calibration frames, common mode correction was performed through identifying correlated pixels and subtracting their shared median values. A combined histogram of all corrected ADU values was constructed, from which an appropriate noise threshold was determined to produce a binary map which differentiated signal from noise. From a selected subset of frames, connected signal pixels were identified and counted to estimate the number

of clusters per frame. Only clusters of connected pixels which satisfy a minimal area threshold were considered to be electron puddles. The total number of such puddles provides an estimate on the average electron dose per frame. For each pixel, all detected events are then ranked by their ADU values from the largest to smallest. With the expected total number of electron counts across the subset of frames, the event at this rank and the next event with a lower rank are selected. The gain threshold for each pixel is then determined from the mean ADU value of both events.

The experiment conducted to compare ReCoDe's on-the-fly calibration and the fine calibration was as follows. A series of flat-field illuminated data frames were collected with a specified electron beam magnification. The dataset was processed using both calibration methods and the average electron dose was recorded. The same process was then repeated at varying electron beam magnifications since the incident electron dose scales inversely with the square of beam magnification. The change in electron counts against the incident dose, which we fitted to a logarithmic model Eqn. (1), with FP as the number of false positives, m as the power term for beam magnification and ρ_0 as the counts at the asymptotic limit of zero magnification, allows us to compare between the two calibration methods.

$$ln(Counts) = ln(FP) + ln(1 + \frac{\rho_0 A}{FP + Mag^m}) \tag{1}$$

We also collected datasets using DE-16's electron counting mode, following the same electron beam magnifications. These counts were also fitted to the same model to compare the differences between the three methods. The curves fitted and the corresponding values for the fitting parameters are stated in Supplementary Table 3.

The fine calibration routine resulted in an approximate 30% decrease in identified electron counts, compared to ReCoDe's fast calibration. As shown in Supplementary Figure 14, the main contributing factor is the area constraint imposed when identifying valid events during the estimation of the average electron dose, which was subsequently used to calibrate each pixel's gain response. The correction for common-mode in the dataset did not significantly affect the final estimated electron counts. The difference in counts was likely due to events that were incorrectly identified as a signal during the initial counting when estimating the average dose per frame. These results suggest that the on-the-fly calibration routine might be more lenient, keeping a larger number of electron events at the cost of misidentifying some noise fluctuations as electron events. Nevertheless, a more stringent recalibration process can always be implemented post reduction for L1 to L4 reduction, if required.

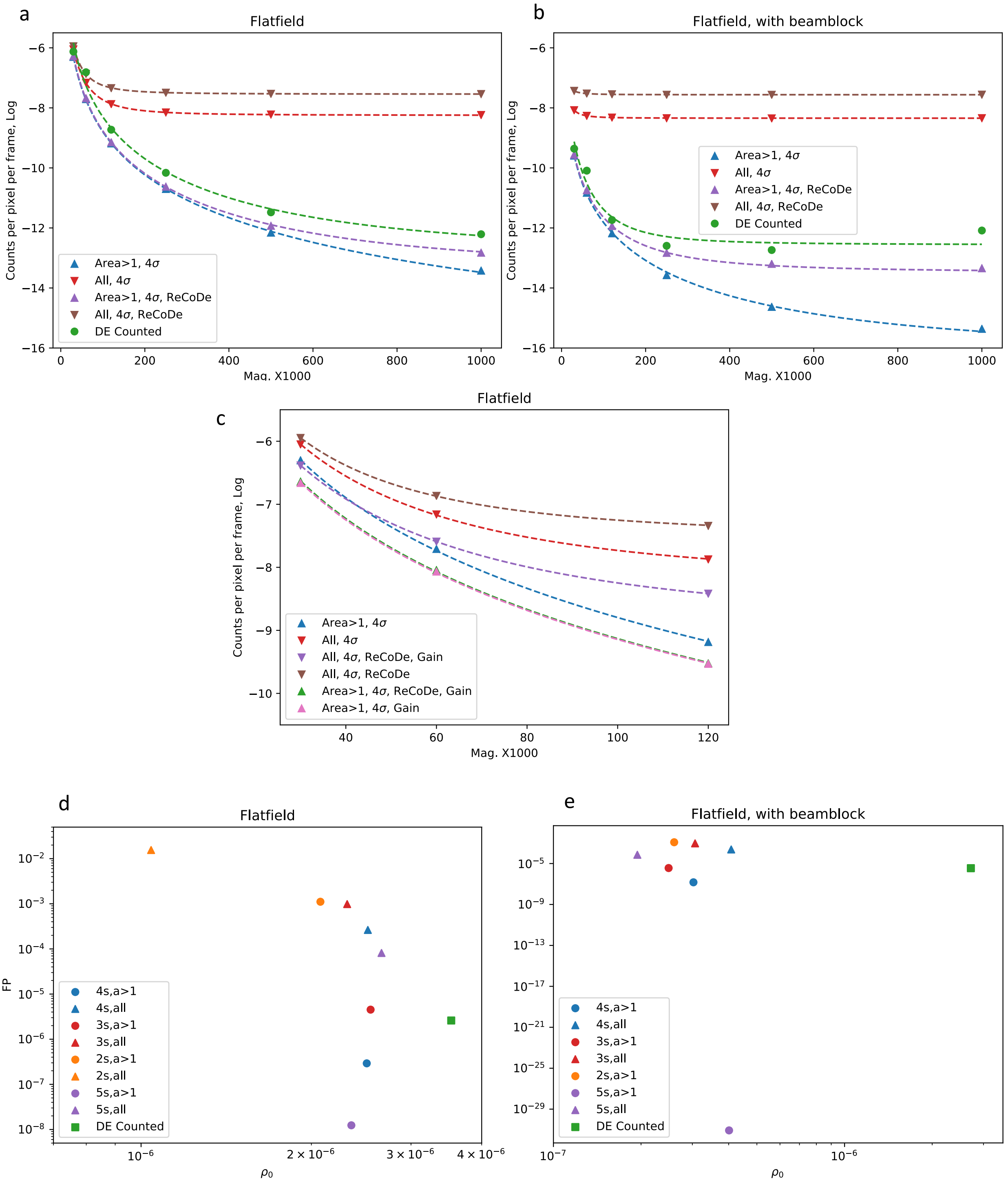

Supplementary Figure 14: Evaluation of on-the-fly and fine calibrations. (a) and (b) Estimated electron counts for varying electron beam magnifications. The average electron dose, with an intensity threshold of 4 standard deviations from the mean noise ADU value, was estimated for different magnifications of electron beam, with and without an area constraint of more than 1 pixel on the electron puddle size. The average electron count, including those obtained through DE-16's electron counting, follows an inverse relationship with the square of the beam magnification. The counts estimated with the area constraint matches the DE counts much closely, compared to without the constraint. Comparatively, estimated average electron dose from ReCoDe's fast calibration follows the same trend only when the magnification was low, diverging when the magnification increased. With the beamblock, DE counts approached an asymptote at high magnification while estimated counts with the area constraint continued to follow a decreasing trend similar to before. Estimated average electron dose with ReCoDe's fast calibration similarly plateaus at high magnification, albeit having a lower asymptotic limit than DE counts. The fitted curves

and the corresponding fitting parameters are listed in Supplementary Table 3. (c) Electron counts after per pixel gain calibration, for varying electron beam magnification. After calibrating for each pixel's gain response, following the fine calibration routine resulted in a ~30% decrease in identified electron events, as compared to ReCoDe's fast calibration routine. The main factor contributing to the reduction in counts is the area constraint imposed on each event, which can significantly reduce the number of false positives. Comparatively, correcting for the common mode did not result in a significant change in the number of counts. These results suggest that ReCoDe's calibration routine is more lenient, allowing a number of noise fluctuations to be misidentified as electron events. Nevertheless, a more stringent recalibration process can be implemented post reduction for L1 to L3 reduced data, if required. This is definitely preferable compared to the alternative where strict constraints are implemented during the early calibration process of the reduction and compression pipeline, which will make it extremely difficult to recover potential events that have been removed. This suggests that there could be further room for data compression if a lower true positive rate can be accepted. (d) and (e) Number of false positives and electron counts at the asymptotic limit of zero magnification, ρ_0. Imposing an area constraint significantly reduced the number of false positives in the estimation of average electron dose, as compared to accepting all identified events including those which only occupy a single pixel. As the intensity threshold increases, the number of false positives decreases consequently, since the number of misidentified events contributed by noise decreases. Unfortunately, this comes at the cost of sacrificing true electron events with low energies, as reflected by the parameter ρ_0, the theoretical maximum for the number of true electron events. Therefore, an appropriate threshold is required to achieve the best compromise, allowing for high true positive rates while maintaining low false positive rates.

Supplementary Table 3: Fitting params used for evaluating on-the-fly and fine calibrations

$$\ln(\boldsymbol{Counts}) = \ln(\boldsymbol{FP}) + \ln(\mathbf{1} + \frac{\boldsymbol{\rho_0 A}}{\boldsymbol{FP} \times \boldsymbol{Mag^m}})$$

A: 921600 px

	FP	m	ρ_0
Area > 1, 4$\boldsymbol{\sigma}$	$1.3(4)\times10^{-7}$	$2.080(2)$	$2.32(2)\times10^{-6}$
All area, 4$\boldsymbol{\sigma}$	$2.617(5)\times10^{-4}$	$2.060(4)$	$2.49(3)\times10^{-6}$
Area > 1, 4$\boldsymbol{\sigma}$, ReCoDe	$1.52(8)\times10^{-6}$	$2.09(1)$	$2.5(2)\times10^{-6}$
All area, 4$\boldsymbol{\sigma}$, ReCoDe	$5.30(1)\times10^{-4}$	$2.04(1)$	$2.3(1)\times10^{-6}$
DE Counting	$3(2)\times10^{-6}$	$2.1(2)$	$4(3)\times10^{-6}$

With beamblock

A: 178161 px

	FP	m	ρ_0
Area > 1, 4$\boldsymbol{\sigma}$	$1.0(3)\times10^{-7}$	$1.87(2)$	$2.2(2)\times10^{-7}$
All area, 4$\boldsymbol{\sigma}$	$2.378(4)\times10^{-4}$	$1.90(9)$	$2.6(8)\times10^{-7}$
Area > 1, 4$\boldsymbol{\sigma}$, ReCoDe	$1.39(4)\times10^{-6}$	$1.87(3)$	$2.3(3)\times10^{-7}$
All area, 4$\boldsymbol{\sigma}$, ReCoDe	$5.20(3)\times10^{-4}$	$1.8(4)$	$2(2)\times10^{-7}$
DE Counting	$4(1)\times10^{-6}$	$2.2(6)$	$3(7)\times10^{-6}$

Supplementary Note 13: Estimating Backscattering

We estimated backscattering ratio by comparing simulated primary and backscattered electron events with actual data. The simulation model employed contains three main parameters, namely the total number of events per frame, the ratio of primary-to-backscattered events and the distribution model for nearest distance between a backscattered and a primary event. For each simulation, the total number of events per frame is fixed following the event counts obtained experimentally. The ratio of primary-to-backscattered events then determines the number of simulated backscattered events in each frame. The distance between a backscattered event and its nearest primary event is assumed to follow an exponential distribution, with the sum of the location parameter and the inverse of the lambda parameter as the mean nearest-neighbour distance for each backscattered event.

Assuming primary events are uniformly scattered across the entire frame, a subset of them are randomly selected as the neighbours of backscattered events. Each backscattered event is then randomly placed beside its primary counterpart, at a distance sampled from the exponential distribution. The nearest-neighbour distances are then calculated for all simulated events and tabulated into a histogram to be compared with the nearest-neighbour distance histogram from the actual electron events. A constraint of nearest-neighbour distances to be larger than two pixels was applied on the simulated events to match the experimental events, propagated from the minimum area constraint on electron puddles. A two-sample Kolmogorov–Smirnov (K-S) test between distributions of nearest neighbour distance for simulated and experimental data to obtain the D-statistic which quantifies the difference between two histograms. The minimum value for D-statistic among 100 repeated simulations for each parameter pair are plotted, and the optimal values are in the range of 8 to 9 (~8.6) for the primary-to-backscattered ratio, and 6 to 7 pixels (~6.4) in average nearest-neighbour distance for a backscattered event.

a

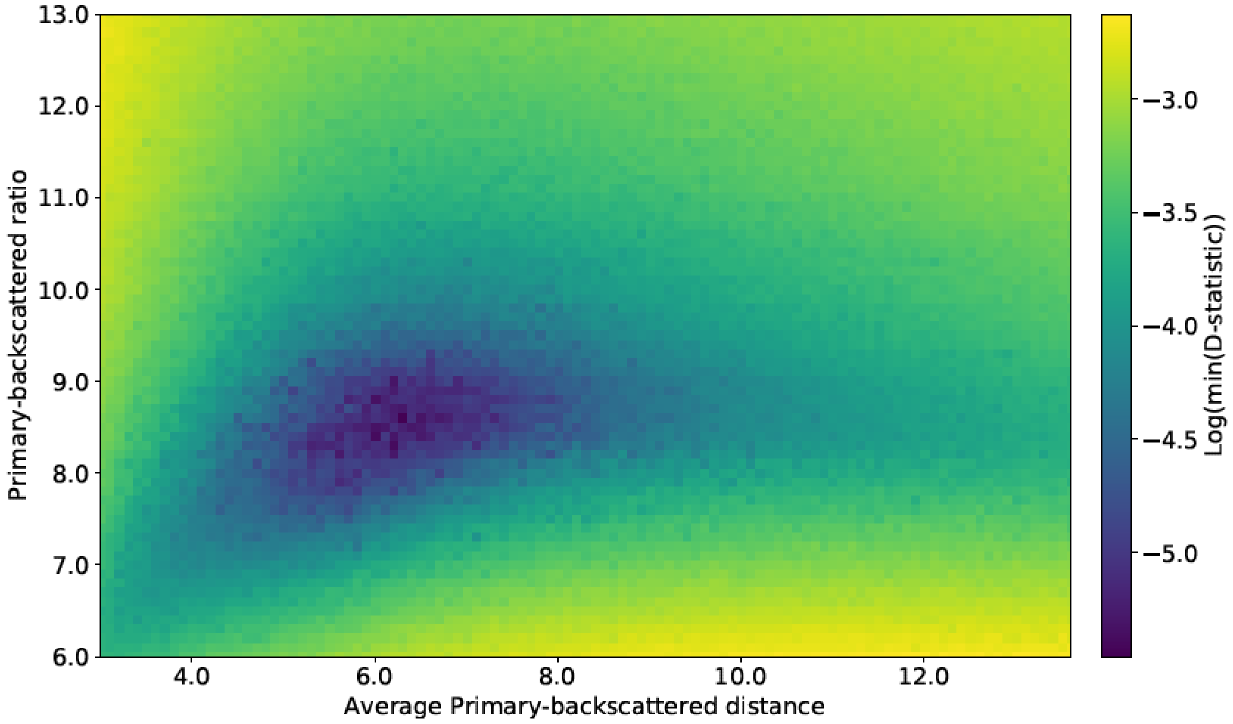

b

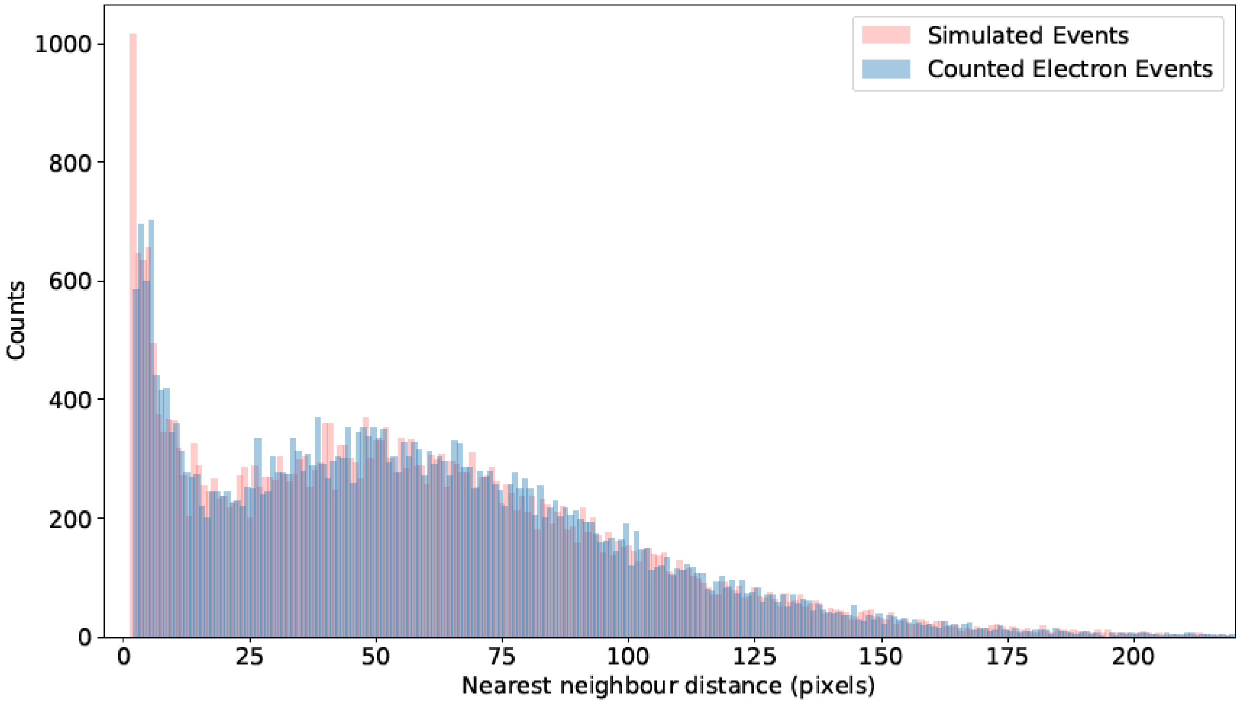

Supplementary Figure 15: Estimating backscattering ratio by modelling primary and backscattered electron events with simulations. (a) The minimum value for D-statistic among 100 repeated simulations for each parameter pair. The optimal values are in the range of 8 to 9 (~8.6) for the primary-to-backscattered ratio, and 6 to 7 pixels (~6.4) in average nearest-neighbour distance for a backscattered event. (b) Histograms of nearest neighbour distances between electron events for both simulated and experimental counted primary and backscattered events. The simulated primary and secondary events are generated following the parameters, a ratio of 8.7 for primary to backscattered events per frame, and 6.4 pixels for the mean nearest-neighbour distance for a backscattered event. For both simulated and experimental dataset, the nearest-neighbour distances are first calculated for all events in each frame. The total number of counts for each distance are then accumulated across all frames, and finally

tabulated into a histogram for comparison. These parameter values gave a combined q-value, following Fisher's method with 100 simulations, of more than a significance level of 0.01, indicating that both histograms were sampled from the same distribution. Comparing the simulated and experimental histograms, K-S test gave a D-statistic of 0.00607, with a corresponding p-value of 0.587, after the constraint of minimum nearest neighbour distance of two pixels was applied.

References

1. Amdahl, G. M. Validity of the single processor approach to achieving large scale computing capabilities. *Proceedings of the April 18-20, 1967, spring joint* (1967).
